# Overview of ongoing cohort and dietary studies in the Arctic

**DOI:** 10.3402/ijch.v75.33803

**Published:** 2016-12-13

**Authors:** Pál Weihe, Peter Bjerregaard, Eva Bonefeld-Jørgensen, Alexey Dudarev, Jónrit Halling, Solrunn Hansen, Gina Muckle, Therese Nøst, Jon Øyvind Odland, Maria Skaalum Petersen, Arja Rautio, Anna Sofía Veyhe, Maria Wennberg, Ingvar Bergdahl

**Affiliations:** 1 Department of Occupational Medicine and Public Health, The Faroese Hospital System, Torshavn, Faroe Islands; 2 National Institute of Public Health, University of Southern Denmark, Odense, Denmark; 3 Centre for Arctic Health, Department of Public Health, Aarhus University, Aarhus, Denmark; 4 Northwest Public Health Research Center, St. Petersburg, Russia; 5 Department of Community Medicine, Faculty of Health Sciences, University of Tromsø - The Arctic University of Norway, Tromsø, Norway; 6 École de Psychologie, Centre de Recherche du CHU de Québec, Université Laval, Québec, Canada; 7 Thule Institute, University of Oulu, Oulu, Finland; 8 Department of Biobank Research, Umeå University, Umeå, Sweden

**Keywords:** Cohorts, Diet, Arctic, Norway, Finland, Sweden, Russia, Faroes

## Abstract

This article gives an overview of the ongoing cohort and dietary studies underlying the assessment of population health in the Arctic. The emphasis here is on a description of the material, methods and results or preliminary results for each study. Detailed exposure information is available in an article in this journal, whereas another paper describes the effects associated with contaminant exposure in the Arctic. The cohort descriptions have been arranged geographically, beginning in Norway and moving east to Finland, Sweden, Russia and the other Arctic countries and ultimately to the Faroe Islands. No cohort studies have been reported for Alaska or Iceland.

## The MISA Study

The Northern Norway mother-and-child contaminant cohort study – the MISA Study – is a cross-sectional study with longitudinal aspects aimed at establishing a new Northern Norway mother-and-child contaminant cohort study. The MISA database is considered suitable for exploring associations between contaminant exposure and diet, enhancing the understanding of the interplay between physiological changes that occur in mothers and contaminant pharmacokinetics (including transfer to the infant before and after birth), and conducting prospective health studies of the children.

Recruitment for the MISA Study began in May 2007 and continued for the next 25 months (until June 2009). A total of 515 eligible women were enrolled in early pregnancy, with 391 women completing the study protocol that included a self-administrated food frequency questionnaire (FFQ) and donation of biological samples for contaminant analysis in the second trimester of pregnancy, just after delivery and 6 weeks postpartum. Daily dietary intake was converted to energy intake, and estimates were made of macro- and micronutrients ingested. Some of the MISA findings were compared to data available in the Medical Birth Registry of Norway (MBRN). Compared with all 2004–2006 mothers in Northern Norway, the MISA Study women were about 2 years older and smoked less. Parity, gestational age and birth weight of the newborns were comparable. The estimated average dietary intake of 8.1 MJ per day was less than that recommended by the Nordic Nutrition Recommendations, but the intake of micronutrients per MJ complied.

Although the final MISA cohort sample size was less than targeted, the generally good comparisons observed between MBRN-registered information for the study cohort and dropouts suggest that this introduced minimal bias. Agreement between the demographic and clinical characteristics of the cohort women and newborns with all births in Northern Norway implied acceptable external validity. The dietary findings also aligned well with Norwegian national data and guidelines and other studies, as did the high prevalence of breastfeeding (1).

Meconium was analysed to establish whether it could be used to predict foetal exposure to organochlorines (OCs) and hydroxylated polychlorinated biphenyls (PCBs). A subset of 40 meconium samples and complementary maternal serum were selected. Multivariate linear regression modelling confirmed that maternal serum was the most consistent predictor of meconium concentrations, with gestational age and time of meconium sampling improving the models. Although more challenging to analyse, the lipid-adjusted OC concentration in meconium appears to be a sensitive and informative foetal exposure index when taking into consideration gestational age and its postpartum sampling time (2).

In a subset of 211 pregnant women, maternal blood concentrations of selected essential and toxic elements in the second trimester of pregnancy were compared with concentrations 3 days and 6 weeks postpartum. Ten selected elements (arsenic, As; cadmium, Cd; cobalt, Co; copper, Cu; lead, Pb; manganese, Mn; mercury, Hg; molybdenum, Mo; selenium, Se; and zinc, Zn) featured three general but distinct concentration patterns across the three collection periods: a progressive increase, a V-shaped curve with a minimum at day 3 and an inverted V-shaped curve with a maximum at day 3 (3).

In a subset of 50 women, blood concentrations of common OCs in the second trimester of pregnancy were compared with concentrations 3 days and 6 weeks postpartum. Both lipids and wet-weight OC levels peaked at birth and were lowest at 6 weeks postpartum. However, this peak was no longer evident when the OC concentrations were lipid-adjusted. Wet-weight OC concentrations appear to be driven by the physiological lipid profiles and are interpreted to constitute biomarkers of lipidaemia. This observation may have implications for the biomonitoring of individuals at risk of type 2 diabetes. Both age and parity were strong predictors for the OCs measured, but no consistent association with body mass index (BMI) was evident. Independent of lipid adjustment, all compounds were positively and significantly correlated with each other (within and across the three collection periods). The peak in OCs during pregnancy suggests that the period spanning the last weeks of the third trimester and the early postpartum days constitutes an optimum sampling window purely from the analytical perspective (4).

## The Tromsø Study

Human exposure to both the legacy and newer persistent organic pollutants (POPs) has changed over past decades. Exposure routes have been largely dietary for the legacy POPs, whereas other routes have also been important for the newer POPs. The legacy POPs have often been observed to increase with age in cross-sectional studies, and this association probably reflects birth-cohort differences in duration and intensity of exposure to these compounds. For the newer POPs, associations with age are not consistent.

The Tromsø Study is a population-based health survey initiated in 1974 to investigate the reasons for high mortality related to cardiovascular disease in Northern Norway. Six surveys have been undertaken since 1974, and the health research topics included have increased. A total of 40,051 people have participated in at least one survey, and 15,157 have participated in three or more surveys. The Tromsø Study was also used to explore the changes in POP concentrations from 1979 to 2007 on an individual basis with a repeat measurement design. Serum samples were obtained from the freezer archive for 54 men who participated in all of the survey points: 1979, 1986, 1994, 2001 and 2007. The archived serum samples were analysed for PCBs, chlorinated pesticides and per-and polyfluoroalkyl substances (PFASs).

Median summed concentrations of PCBs and OC pesticides decreased by 22% (1979–1986), 52% (1979–1994), 54% (1979–2001) and 68% (1979–2007) (5). The concentrations of dichlorodiphenyltrichloroethane (DDT) and dichlorodiphenyldichloroethylene (DDE) decreased from 1979, whereas most PCBs decreased from 1986. The median summed PFAS burdens increased from 1979 to 2001 and then decreased from 2001 to 2007 (6). The results indicate that concentrations have followed calendar year trends rather than “increasing with age” per se, and concentrations have followed birth-year-dependent patterns over time. Furthermore, the composition of POPs in serum has changed over the almost 30-year period. Assessments of age, calendar year and birth-cohort trends showed that calendar time was the dominant influence.

The concentrations and time trends of four PCBs were compared to predicted concentrations from a human exposure mechanistic model (5). The predicted and measured concentrations, time trends and birth-year patterns were in good agreement and so inspire confidence in such models.

The trends observed between 1979 and 2007 probably reflect overall trends in the use and emission of the different POPs, together with compound-specific persistency, bioaccumulation potential and long-range transport. Study design and population characteristics must also be considered in monitoring studies. The Tromsø Study has increased knowledge of intra-individual variation in POP concentrations with respect to time and exposure history, which is essential for understanding past exposure and predicting future exposure. The findings have important implications for future studies of exposure and vulnerable groups (5).

## Northern Finland 1966 Birth Cohort

Information is available on individuals born into the Northern Finland 1966 Birth Cohort in the provinces of Oulu and Lapland since the 24th gestational week as well as on their mothers and, to a lesser extent, fathers. A total of 12,058 live-born children were born into the cohort (around 96% of all eligible), and 11,665 were alive in 1997. Data were collected by questionnaires, from hospital records and various registers and databases (social benefits, medication reimbursement, hospital discharges and deaths, community wealth), as well as by interview and clinical examination at birth, at age 1 and 14 years and at age 31 years when a comprehensive follow-up was conducted on each subject's well-being, social standing and health. In 1997, all members of the cohort who lived in the provinces of Oulu and Lapland (n=7,191) or who had moved to the capital region (n=1,272) completed a questionnaire and underwent a health examination. Those living in other parts of the country (n=2,164) or abroad (n=695) were also sent the questionnaire. In total, 8,676 people returned the questionnaire, making a response percentage of about 77%. About 71% attended the health examination. Selected blood samples (n=250) from the 1997 sampling were analysed for toxic and essential elements to establish levels in persons born and living for the last 5 years in the eastern and western part of Lapland.

## The Northern Sweden Health and Disease Study

The Northern Sweden Health and Disease Study (NSHDS) consists of three study cohorts in northern Sweden: the Northern Sweden MONICA Study, the Västerbotten Intervention Program (VIP) and the Mammography Screening Program (MSP).

The Northern Sweden MONICA Study was originally part of the multicentre WHO survey MONICA (Multinational Monitoring of Trends and Determinants in Cardiovascular Disease). In the Northern Sweden MONICA Study, 2,000 or 2,500 randomly selected participants aged 25–75 years (25–65 years before 1994) have been invited to the health survey every fourth or fifth year since 1986. Seven surveys have been conducted to date, and 11,800 individuals have participated, of whom 3,500 have participated more than once. Participants undergo a medical examination focusing on risk factors for cardiovascular disease and complete an extensive questionnaire on lifestyle factors, including a FFQ. Participants are asked to donate fasting blood samples to be stored in a biobank for future research. Urine samples were collected from a proportion of the participants in the surveys from 2009 to 2014.

The VIP began in 1985 and was designed after the MONICA Study. In addition to the health examination, questionnaires and collection of blood samples to the biobank, VIP includes an intervention in which participants are offered counselling regarding lifestyle modifications with a trained nurse. The inhabitants in the county Västerbotten are invited to the intervention the year when they become 40, 50 and 60 years old (until 1994 also 30 years old). By March 2015, 98,300 individuals had participated with 36,100 having participated more than once. No urine has been sampled.

The MSP was conducted between 1995 and 2006. Women 40–70 years of age were invited to mammography screening and were asked to donate blood samples to the biobank. Limited information on lifestyle factors was collected. In total, 28,800 women have participated with 14,600 having participated more than once.

The stored blood samples make prospective studies of the environmental contaminants possible within the NSHDS.

Environmental contaminants have been analysed in subgroups of the Northern Sweden MONICA Study. Concentrations are available for Cd and Pb in blood for 1990–2014 and Hg for 1990–2009. In the latest survey, from 2014 some organic pollutants were analysed in urine (10 phthalates, bisphenol A, bisphenol F, hydroxypyrene, triclosan, pesticides, trichloropyridinol and 3-phenoxybenzoic acid). Bisphenol A was also analysed in urine in 2009.

Various case–control studies concerning health effects of environmental pollutants have been conducted within the NSHDS. For example, those on Hg in relation to myocardial infarction and stroke; Cd, Pb and Hg in relation to kidney diseases; Cd in relation to fractures; Cd and Pb in relation to B-cell malignancies; and data from NSHDS are included in international collaboration on POPs and various cancers. Case–control studies concerning exposure to Cd and POPs and risk of diabetes are ongoing.

There are plans to create an environmental contaminant cohort within the NSHDS. This will include individuals for whom analyses of metals and POPs have been carried out.

## Chukotka dietary and exposure study

Data were collected in 2001–2003 in Chukotka (Russia) on PCB and DDT contamination of different local foods and indoor materials. Exposure of indigenous people was evaluated by comparing pollutant levels in foods with levels in human blood in people living in coastal and inland regions (7–9). Because DDT degrades to DDE, the ratio of *p*,*p*′-DDE to *p*,*p*′-DDT is commonly used in environmental epidemiology practice as a measure of the remoteness of DDT exposure events: the higher the score, the lower the concentration of the original DDT and the longer the exposure.

The DDE/DDT ratios in Chukotka fresh local foods vary widely. High ratios were found in whale meat (up to 16), seal meat (up to 27) and bearded seal fat (up to 65), which indicate little fresh contamination, whereas low DDE/DDT ratios were observed in walrus meat (up to 1.5), walrus fat (up to 8.5) and bearded seal meat (up to 6.2). As well as exposure, these widely varying ratios could reflect differences in nutritional habit (bearded seals and walrus feed on benthic invertebrates, whales feed on krill and seals feed on fish), variability in DDT metabolism (still poorly studied) and DDE/DDT accumulation/elimination processes in these large marine animals with a thick layer of subcutaneous fat. Fish (migratory and freshwater), poultry and venison are characterized by low DDE/DDT ratios [1–5], which could indicate “fresh” exposure. Extremely low DDE/DDT ratios in washouts and scrapes from the walls inside dwellings (0.4) are conclusively linked to the use of “fresh” DDT as a household insecticide (9).

Comparison of DDE/DDT ratios in the food and blood of aboriginal Chukotka people suggests that the higher ratios in marine mammals are responsible for the higher ratios in the blood of coastal natives, and the much lower ratios in reindeer meat, poultry and fish are responsible for the lower blood ratios for inland residents.

Despite considerable variation, the indigenous coastal residents have a blood DDE/DDT ratio [18–19] that is almost double that of their inland neighbours [11–12], which indicates a substantial amount of relatively “fresh” DDT contamination in the inland regions (10–15% vs. 4–6% of 4,4′-DDT, respectively). This implies that marine food-chain DDT has a more “long-standing” global origin than terrestrial food-chain DDT (8).

Blood PCB congener “composition” in indigenous coastal residents shows a low percentage of low-chlorinated and dioxin-like PCB congeners and a large share (up to 60%) of “triad” congeners (PCB128–PCB138–PCB153). Inland residents are characterized by a much higher proportion of low-chlorinated and dioxin-like congeners and a lower share of “triad” congeners (35–38%). The composition of PCB congeners in the blood of inland residents differs from that of inland local foodstuffs, unlike the coastal residents whose blood PCB “formula” is very similar to that of marine mammal tissues. There was no similarity between the PCB congener structure of household indoor materials (washouts and scrapes from the walls) and the blood of natives (9).

## Follow-up Chukotka coastal mother–child study 2001–2007: exposure and infection disease study

Levels of persistent toxic substances (PTS) in blood from 17 mothers and cord blood from the corresponding 17 babies born in the Chukotka coastal area in 2001–2002 were compared with PTS levels in blood sampled from the same women and their 5-year-old children in 2007 with the aim of examining the influence of breastfeeding on maternal POPs serum levels and the link between children's POPs blood levels and the frequency of infectious diseases (10,11).

Maternal blood levels of POPs decreased significantly during the 5-year period (by 33–74%), blood Pb levels decreased by 21% and blood Hg levels remained the same. The infant blood serum levels of most POPs increased considerably over this period, while blood Pb levels were unchanged and Hg levels decreased by 31%.

Results showed that, during the 5-year period, maternal PCB levels became similar to those observed in cord blood in 2001 and vice versa – infant PCB levels in 2007 became similar to maternal levels in 2001. The ratio of PCB congeners in blood of puerperia–mother and foetus–child in coastal Chukotka was unchanged 5 years after the first examination, which indicates that elimination/accumulation rates for the various congeners were effectively the same both in mothers and children. The average elimination half-life of PCB congeners PCB105–PCB187 in maternal blood was 4–6 years, and for total PCB was 5.7 years.

The maternal DDT metabolite levels fell by 70% each, whereas the DDE/DDT ratio stayed at about 12, which could indicate “old” sources of exposure. The infant DDE/DDT ratio increased by 84%, from 10.6 to 19.5.

On average, the medical files showed 4.8 infectious disease occurrences per child per year (range 0.9–9.6). For each individual child, there was no significant association between the annual number of infectious diseases (2001–2007) and POPs cord blood serum concentrations (2001). An interesting observation was that two children maximally exposed (to POPs and metals) got sick less often than others (10,11).

## Chukotka birth cohort 2001–2003: exposure and reproductive effects

In 2001–2002 in Chukotka delivery departments, 126 indigenous pregnant women (68 coastal and 58 inland) were interviewed and gave blood samples. Births with adverse outcomes were observed in almost every fourth woman (23%): premature births (22 cases) partially accompanied by low birth weight (13 cases), stillbirths (3 cases) and congenital malformations (3 cases – a heart defect and two multiple defects, one stillborn).

Negative (but insignificant) associations were found between high POPs levels and premature births and low birth weight. No associations were found for metals. Average POPs blood levels among mothers with preterm births were lower than those with a normal length of gestation: PCBs by 13–16%, hexachlorocyclohexane (HCH) by 14%, oxychlordane by 43%, ΣDDT by 11% and Mirex by 33%.

Maternal POP blood levels were higher in cases of stillbirth: ΣPCBs by 27%, 4,4′-DDE by 84%, 4,4′-DDT by 304%, ΣDDT by 94% and hexachlorobenzene (HCB) by 20%, but the differences were not statistically significant.

Maternal POP blood levels were also higher in cases of congenital malformation: ΣPCB by 156%, DDT metabolites by 38–54%, HCB by 119%, ΣHCH by 95% and oxychlordane by 575%. These associations were statistically significant for oxychlordane (p=0.04) and HCB (p=0.02) only.

In terms of POPs and the sex ratio of newborns (70 boys and 56 girls), women with higher exposure to PCBs and other POPs gave birth to girls more often than boys. On average, PCB blood levels among mothers of boys were 13–30% lower than for mothers of girls, and for other POPs 18–44% lower. The differences were statistically significant for ΣHCH (p=0.033) and 4,4′-DDT (p=0.042). Average levels of metals in the blood of mothers having boys and mothers having girls were similar.

Higher POPs blood levels were noted among women with earlier menarche, shortened menstrual cycle and prolonged bleeding (12).

## Kola Lapland 2001–2006: POPs and diabetes mellitus

In 2006, and under the framework of the International Barents Secretariat project “Revealing the hidden diabetes mellitus in Lovozero district of Murmansk Oblast,” 4,359 residents of Kola Lapland in Murmansk Oblast were interviewed and had their blood glucose levels analysed (2,736 rural and 1,623 urban, including Sami – 694, Komi – 910 and Nenets – 80). This showed that the risk of type 2 diabetes (overweight/obesity, enhanced blood pressure, sedentary lifestyle, malnutrition and alcohol abuse) was three- to sevenfold lower in indigenous residents than non-indigenous residents. Signs of diabetes were absent among Sami people of the remote villages. Elevated blood glucose levels were found mainly in large settlements. Indigenous residents in remote villages demonstrate minimum risk for diabetes mellitus, and this may be related to their traditional diet based on local foods, a physically active lifestyle and minimal consumption of high carbohydrate foods.

Data collected as part of the Russian Arctic PTS study (2001–2003) on blood POPs levels in indigenous residents of Kola Lapland (83 residents had blood sampled for PTS analyses) gave an opportunity to compare the results of both projects.

A comparison of diabetic status and POPs exposure showed that overweight/obesity, high blood glucose and type 2 diabetes diagnoses were associated with higher blood levels of POPs (ΣPCB, DDTs, ΣHCH and HCB), although this was not statistically significant (7).

## Nunavik Child Development Study

The Nunavik Child Development Study (NCDS) is a prospective mother–child cohort study taking place in Nunavik (Arctic Quebec), Canada. It was designed to extend previous findings on the effects of OCs, Hg and Pb on child health and development by following up a sample of mother–child dyads that were found to be prenatally and postnatally exposed to high levels of POPs and heavy metals. The primary objective of the NCDS was to document the growth, neurobehavioral and cardiac effects of pre- and postnatal exposure to OCs with endocrine-related disruptive properties, as well as to Hg and Pb. A secondary aim was to test whether nutritional variables (e.g. prenatal polyunsaturated fatty acid intake, breastfeeding and childhood vitamin deficiency) mediate and/or mitigate the effects of environmental contaminants on health and development. The NCDS was made possible by the Nunavik Cord Blood Monitoring Program, through which exposure to environmental contaminants was determined from umbilical cord blood samples obtained from almost all Nunavik infants born between 1994 and 2001 (13,14). Between November 1995 and March 2001, pregnant Inuit women from the three largest communities of the Hudson Bay coast were invited to participate in a first follow-up (15), which allowed the testing of 190 infants at 6 and 12 months of age. Between 1999 and 2001, 110 preschool-aged children who had not participated in the follow-up during infancy underwent neuromotor and neurophysiological testing (16,17). Between 2005 and 2010, 294 children of 11 years old were tested, whether or not they had been assessed during the infancy or preschool periods. The cohort is currently being followed up at adolescence (2013–2016).

Mothers who had volunteered to provide cord blood samples at the birth of their child under the Cord Blood Monitoring Program were contacted by phone, provided with information about the study protocol and invited to participate with their children in the NCDS. Inclusion criteria were children of 8.5–14.5 years in age, a birth weight of 2.5 kg or above, a gestation duration of 35 weeks or more and the absence of either birth defects, neurological or health problems, or pervasive developmental disorders. Over a 5-year period (September 2005 to February 2010), 294 children and their mothers participated in neurocognitive assessments in the three largest villages in Nunavik. Participants residing in other communities were flown by plane to one of the three villages selected for testing. Children were assessed to establish physical growth, vision, intellectual function and cognitive development, behavioural development, and heart rate and blood pressure. A maternal interview was conducted to provide information on demographic background, lifestyle during pregnancy and family environment. Cord blood samples collected at birth and child blood samples collected at the time of testing were analysed for chlorinated pesticides, PCBs, Hg, Pb and total plasma lipids. Many potentially confounding variables and effect modifiers were assessed through the blood biomarkers, medical files and maternal self-reporting.

## Inuit adult cohorts: Canada

The Nunavik Health Survey Qanuippitaa was conducted between 30 August and 1 October 2004 on the scientific research vessel CCGS Amundsen. The research group visited the 14 communities of Nunavik (www.qanuippitaa.com) and recruited 917 participants. Samples were analysed for POPs that had also been determined during the 1992 Santé Québec Health Survey. Plasma concentrations of new halogenated hydrocarbons such as polybrominated diphenyl ethers (PBDEs), perfluorooctanesulfonate (PFOS), hydroxy-PCBs, methyl sulfone PCBs and chlorophenols were also determined.

The Inuit Health Survey (2007–2009) was a comprehensive study that included the measurement of dietary intake of contaminants, contaminant body burden, as well as other determinants of health and their relationship with health outcomes of the participants. It was the first time that such a complete set of data had been collected from Inuit in Nunavut, the Inuvialuit Settlement Region and Nunatsiavut. The study was the result of the integrated efforts of Inuit, Inuit organizations, the Departments of Health of the Territorial and regional Inuit governments, and scientists from a variety of different disciplines. Of the 2,595 individuals participating in the Inuit Health Survey, 2,172 provided blood samples. The body burden of several metals (e.g. Cd, Pb) and POPs (e.g. PCBs, DDT, DDE, toxaphene, chlordane and PBDEs) were measured for Inuit participants (n=2,172) from 36 communities in Nunavut, Nunatsiavut and the Inuvialuit Settlement Region, in Canada (18–39).

### INUENDO

The INUENDO project: Biopersistent organochlorines in diet and human fertility. Epidemiological studies of time-to-pregnancy and semen quality in Inuit and European populations were supported by the European Union Fifth Framework (FP5) program (40). The cohort was established in 2002–2004 and involved about 1,400 pregnant women from Greenland, Poland and Ukraine, as well as studies on about 600 fertile couples from Sweden (fishermen and their wives). The Greenlandic part of the study was 438 men (average age 31; range 18–72) and 572 pregnant women (average age 27; range 18–42).

The study included measurement of PCB153 and *p*,*p*′-DDE and bioeffect markers of serum legacy POP-induced oestrogen- and androgen-receptor transactivity and epidemiological cross-sectional studies on male and female reproductive health on these individuals in relation to the measured exposures.

The associations between previous exposure to PCB153 and *p*,*p*′-DDE and foetal loss were studied in this cohort. The risk of ever experiencing a foetal loss increased at higher levels of PCB153 and *p*,*p*′-DDE exposure, although the results were inconsistent between countries (41).

The association between foetal exposure to PCB153 and *p*,*p*′-DDE and birth weight and gestational age was also evaluated, indicating lower birth weight and shorter gestational age at higher POP exposure (42). These results were included in a large European meta-analysis of birth weight in relation to PCB and DDE exposure, confirming the negative association between PCB exposure and birth weight (43). However, it has been questioned whether the results could have been biased by maternal weight gain during pregnancy (44).

### CLEAR

The CLEAR project: Climate Change, Environmental Contaminants and Reproductive health was supported by the EU's Seventh Framework Programme for Research (FP7). In addition to modelling the effects of climate change on long-range transport of contaminants, the project includes a series of cross-sectional studies on male and female reproductive health combined with a follow-up study on childhood growth and development at 6–9 years of age in a cohort of about 1,400 mothers, fathers and offspring from Greenland, Poland and Ukraine. The original INUENDO cohort was established in 2002–2004, and the CLEAR follow-up on the children was undertaken in 2009–2012.

In addition to PCB153 and *p*,*p*′-DDE, serum or full blood were analysed for HCB, six PBDEs, one PBB, six phthalate metabolites, eight perfluorinated compounds (PFCs), bisphenol A, cotinine, Pb, Cd and Hg (45,46). The contaminant distribution varied widely between countries with the highest level generally found in Greenland.

### IVAAQ

The Greenland Child Cohort IVAAQ recruited 403 mothers and newborn children in West Greenland during 1999–2005. Several follow-up studies have since been carried out. The IVAAQ study has a strong focus on contaminant exposure but information about lifestyle factors such as diet, physical activity, smoking and alcohol are also part of the study.

### ACCEPT

The aim and perspectives of the ongoing ACCEPT project – Adaptation to Climate Change, Environmental Pollution, and Dietary Transition – are to establish a geographical Greenlandic mother–child cohort (47). The overall aim was to obtain a cohort compatible with international and especially other circumpolar child cohorts. ACCEPT will explore possible health outcomes of lifestyle and dietary changes during a period of rapid global change, particularly climate change. Being a part the FETOTOX (http://fetotox.au.dk) international network of mother–child cohort studies (Norway – the MISA study; Denmark; and Shanghai, China) carried out with similar protocols, ACCEPT will compare contaminant-related health effects between populations in several countries with regard to differences in exposure patterns, genetics and lifestyle factors.

The ACCEPT study has established a prospective mother–child cohort designed to determine exposure to environmental contaminants during pregnancy, and the development of the foetus and child as well as health effects later in life in the Greenlandic population. ACCEPT is being undertaken in cooperation with the Greenlandic health care system and the Institute of Nurse and Health Sciences at the University of Greenland, Nuuk. ACCEPT represents a formal and ongoing information service on environmental health issues for local communities in Greenland (health professionals as well as the general population). The ACCEPT study protocol includes the enrolment of women in early pregnancy (before the end of gestation week 13). A questionnaire on lifestyle and food frequency is administered by midwives, and women donate biological samples for a biobank, as well as blood samples for determining contaminant levels and several effect biomarkers in the first trimester and cord blood, and some breast milk before day 6 postpartum. Data on the newborn child are obtained from a perinatal journal maintained by the visiting nurse. The children will be followed up on skills at year 3–4 by telephone interview. The ACCEPT database is considered suitable for exploring associations between POP exposure and diet, providing information on contaminant pharmacokinetics (including maternal transfer to the infant before and after birth), and for prospective child health studies. Recruitment occurred between August 2010 and August 2011, and 192 pregnant women were enrolled. Unforeseen problems prevented recruitment between summer 2011 and summer 2013. Another 395 pregnant women were enrolled during the last 6 months of 2013, 2014 and 2015, increasing the cohort to include 587 mother–child pairs.

In total, 587 pregnant women are enrolled in the ACCEPT cohort: 13 from North (Qaanaaq, Upernavik, Uummannaq); 88 from Disko Bay (Ilulissat, Aasiaat, Qeqertarsuaq, Qasigiannguit); 455 from West (Sisimiut, Maniitsoq, Nuuk, Paamiut); 10 from South (Qaqortoq, Nanortalik, Narsaq); and 21 from East (Tasiilaq, Ittoqqortoormiit). The first data from the 192 pregnant women sampled in 2010–2011 on maternal serum POP levels have been published (47). A trend of higher intake of marine mammals in the eastern and northern regions was reflected by a higher n-3/n-6 fatty acid ratio. Participants in the eastern region also tended to have a higher intake of terrestrial species. A higher percentage intake of seabird species was seen for pregnant women in the western region. Compared to earlier reports, decreased levels of legacy POPs, Hg, Pb, PFOS and perfluorooctanoic acid were observed, but the levels of PFAS congeners perfluorohexane sulfonate and perfluorononanoic acid remained similar to those reported previously.

A substudy on lifestyle, reproductive factors and food intake for the same pregnant women sampled in 2010–2011 showed a relatively higher BMI and high smoking frequency; age and regional differences were found for alcohol consumption, breastfeeding plans and food intake profile (48).

## Follow-up of children from the IVAAQ and CLEAR cohorts in Greenland

In 2010, 223 children from the IVAAQ cohort were followed up for the presence of otitis media by otoscopy, tympanometry and review of hospital records. The hypothesis of a positive association between maternal POP concentrations in pregnancy and increased prevalence of chronic otitis media in children aged 4–10 years was not confirmed in this study (49). Thus, these results contrast with the results obtained for otitis media in the study by Dewailly and Dallaire.

In 2012, 311 children from the IVAAQ (n=198) and CLEAR (n=113) cohorts were followed up to assess the growth pattern and health (assessed by contact with the health care system) among children aged 6–10 years. Children aged 0–18 months had higher weight and height than the reference populations. The study concluded that growth among Greenlandic children older than 18 months corresponded with growth reference curves from Sweden but did not match those from Japan or WHO data (www.who.int/childgrowth/en). On average, the Greenlandic children had 40 contacts with the health care system during a period of 6–10 years. They were hospitalized more often than Danish children, but had fewer contacts with the primary sector (50).

A total of 409 children now aged 7–9 years were invited to a follow-up in 2013–2014 to test the hypothesis that exposure to POPs may negatively affect the immune response to vaccines (diphtheria and tetanus).

## The Greenland population health studies

Cross-sectional countrywide studies of the adult indigenous population in Greenland have been undertaken three times since the early 1990s: 1993–1994, 1999–2001 and 2005–2010. The data were obtained by questionnaire and for most participants also by clinical examination and sampling of biological media. The main themes of the studies were general health and living conditions, mental health, substance abuse including smoking, diet, physical activity, diabetes and cardiovascular disease. All studies included dietary information and some data on contaminants.

In 2014, all 6,008 participants were followed up in disease registers (mortality registry, cancer registry and registry of hospital discharges), and 2,102 participants were re-examined. In a subsample of these participants (n=547), blood samples were drawn and analysed for Hg and selected POPs.

A FFQ with portion sizes was used to create dietary pattern groups (51). The questionnaire was validated against blood Hg, and it was shown that the frequency of meals of marine mammals showed similar associations with blood Hg as did calculated intake in grams (52). The dietary pattern “Traditional food” was positively associated with type 2 diabetes (53). Results from the three population health surveys demonstrated a decreasing trend for PCBs and pesticide levels in blood (54). Neither Hg nor POPs showed any association with blood pressure in Greenlandic Inuit (55,56).

### Birth Cohort 1 in the Faroe Islands

A cohort of 1,022 singleton births was assembled in the Faroe Islands during a 21-month period in 1986 and 1987 (57). Frequent whale meat dinners during pregnancy, frequent consumption of fish (to a much lesser degree) and increased parity or age are associated with high Hg concentrations in cord blood and maternal hair. Mercury concentrations in cord blood correlated moderately with blood selenium. Lead concentrations in cord blood were low (median, 82 nmol/L). Because the effects of foetal childhood exposure to MeHg are persistent, detailed examinations of children with prenatal exposure to this neurotoxicant were performed at age 7 years (1993–1994). A total of 917 of the children (90.3%) completed the examinations. Past medical history, current health status and social factors were recorded on a self-administered form. The physical examination included a functional neurological examination with emphasis on motor coordination and perceptual-motor performance. The main emphasis was placed on detailed neurophysiological and neuropsychological tests that had been selected on the basis of a range of considerations (58,59). A follow-up examination was performed using the same test methods when the cohort members were 14 and 22 years of age. Cohort 1 is currently undergoing a follow-up at the age of 27 years, with special emphasis on how glucose metabolism and immune response are associated with environmental contaminants. The neurobehavioral effects found in Cohort 1 at age 7, 14 and 22 years are described in the article on health effects in this journal.

### Birth Cohort 2 in the Faroe Islands

The findings from Birth Cohort 1 suggested that exposure assessment should encompass several lipophilic pollutants in addition to MeHg. As a follow-up, Cohort 2 was therefore established during a 12-month period in 1994–1995 and included 182 singleton term births from consecutive births at the National Hospital in Tórshavn, Faroe Islands. Relevant obstetric data were obtained by standardized procedures and supplemented by a brief nutrition questionnaire. The geometric mean (GM) for MeHg was 20.96 µg/L in cord blood and 4.13 µg/g in maternal hair. The maximum maternal hair-Hg concentration was 16.31 µg/g. The GM for maternal serum PCB was 1,126 µg/kg lipid with a maximum of 18,446 µg/kg lipid. The children were first examined by the Neurological Optimality Score (NOS) at age 2 weeks (adjusted for gestational age). Subsequent examinations occurred at age 18 months and then at 12-month intervals to age 66 months. At 42 months, a comprehensive medical examination with the NOS was included (60). For comparison with Cohort 1, detailed neurobehavioral tests were carried out at age 7 years. A repeat examination was undertaken at age 10 years. The complete profile of neurobehavioral development has been analysed, and the study suggests that prenatal MeHg exposure may have an adverse effect on Visual Evoked Potential findings despite the absence of clinical toxicity to the visual system. However, this association was apparent only after adjustment for n-3 PUFA status (61). This cohort also participated in a study of serum antibody concentrations as a measure of the effects of routine childhood immunizations, showing that increased perinatal exposure to PCBs may adversely impact on immune responses to childhood vaccinations (62). The clinical implications of insufficient antibody production emphasize the need for prevention of immunotoxicant exposures.

### Birth Cohort 3 in the Faroe Islands

New insight into health risks caused by environmental pollutants and changing exposure patterns in the Faroes led to the formation of Cohort 3 from 656 consecutive births in Tórshavn between November 1997 and March 2000. Following dietary recommendations from the Faroese health authorities, MeHg exposure had now decreased thus allowing better characterization of possible effects of PCBs and other persistent contaminants. Additional attention was turned to the PFCs, as they have been documented as contaminants of marine food chains with possible toxicity to the immune system. Cohort 3 is similar to the two previously generated cohorts in that serum was collected from the mother at the last antenatal examination (34th week of pregnancy). Other samples collected from the mother–child pairs include cord blood and serum, maternal hair at parturition and milk on days 3–5 (before mother and child were discharged) and at 2 weeks. Nutritional habits were recorded by questionnaire. A subgroup of cohort children was examined with regard to immunological parameters at ages 11 and 18 months. The first comprehensive medical examination was carried out just before the booster vaccination at age 5 years, with a follow-up blood sample 1 month after vaccination. The children were examined again at age 7 years, with a main focus on immunological parameters such as formation of antibodies and allergic response (63). The conclusion of this study was that developmental PCB exposure is associated with immunotoxic effects on serum concentrations of specific antibodies against diphtheria and tetanus vaccinations. The immune system development during the first years of life appears to be particularly vulnerable to this exposure. Elevated exposures to PFCs were associated with reduced humoral immune response to routine childhood immunizations in children aged 5 and 7 years (64). A follow-up study at 13 years was undertaken with 533 (86.7%) participants (255 females and 278 males). This follow-up study aimed (a) to describe the most vulnerable age to effects of immunotoxicant exposure on antibody responses to vaccines and further to detect whether these effects continue into adolescence; (b) to investigate whether early postnatal or cumulative exposure to immunotoxicants affect the occurrence during childhood of allergic diseases and raised serum IgE concentrations; (c) to determine whether blood lymphocyte populations and cytokine production elicited by a toxoid challenge are affected by immunotoxicant exposures; and (d) to describe which environmental immunotoxicants are responsible for these effects, and at which exposure levels. The follow-up study also encompassed a thorough clinical examination. The main focus was on development of allergic disease, using International Study of Asthma and Allergies in Childhood (ISAAC) criteria. A questionnaire similar to previous examinations was used and included the child's current health, diet and past medical history with emphasis on infectious and allergic disease, as well as family history for asthma, chronic bronchitis, atopic dermatitis, allergic eczema and rhinitis/pollinosis. Blood samples were obtained for measurements of antibodies and exposure biomarkers. Skin prick tests were also used to detect allergic reactions.

### Birth Cohort 5 in the Faroe Islands

The most recent of the Faroe Islands’ cohort studies began during an 18-month period between October 2007 and April 2009. The total number of mother–child pairs was 501 (70% of the eligible population). Blood was taken from the cord, and blood, hair and milk were obtained from the mother. The analytical results show Hg exposure to have declined substantially, with the GM being 4.8 µg/L cord blood and 0.71 µg/g hair. The maximum maternal hair-Hg concentration was 6.3 µg/g. OC exposure has also begun to decline. The GM for serum PCB was 420 µg/kg lipid, with a maximum of 2,965 µg/kg. In connection with the birth-cohort formation, detailed data were obtained about time-to-pregnancy and obstetric parameters. In addition, 281 fathers participated in an examination of sperm quality along with a blood sample for OC analysis. Both grandmothers were invited to complete a dietary questionnaire and provide blood for OC analyses – as a possible reflection of prenatal exposure of the birth-cohort parents. A total of 343 maternal and 206 paternal grandmothers participated. The children were examined at the age of 18 months. Follow-up includes a questionnaire at 42 months and a clinical examination at 5 years of age, with emphasis on immunological parameters.

## The septuagenarian cohort of the Faroe Islands

To complement the birth-cohort studies, studies are also being undertaken to examine the health status of elderly Faroese residents regarding their lifetime exposure to marine pollutants. A cohort of 1,131 Faroese residents aged 70–74 years was formed and 713 were examined (64% of the eligible population). All subjects underwent a thorough physical examination, with a focus on neurobehavioral and cardiovascular functions, as well as body weight, diabetes and general health. Birth weights were obtained from midwife charts at the Faroese National Archives. Cumulative exposure to major marine pollutants was determined from blood sample analysis. Dietary habits concerning the consumption of traditional food during childhood and adolescence, adulthood, and the most recent year were recorded (65). Information concerning current health and past medical history, including medication, risk factors such as smoking and alcohol use, and body weight at age 20 years was also recorded (66).

Serum 25-hydroxyvitamin D3 (S-25(OH)D3) was measured in 669 of the 713 subjects for whom sufficient serum was available: 19% had S-25(OH)D3 concentrations <25 nmol/L and only 10.3% had S-25(OH)D3 concentrations >80 nmol/L. In a logistic regression analysis, BMI <30 kg/m^2^, blood sampling in summer season, eating pilot whale blubber more than once per month and female sex were positively associated with vitamin D levels of >80 nmol/L. The high prevalence of low vitamin D levels among the elderly Faroese population reflects the low skin synthesis during most months of the year, which is caused by the limited sun exposure and insufficient benefits from marine diet (67).

Septuagenarians with type 2 diabetes or impaired fasting glycaemia tended to have higher PCB concentrations and higher past intake of traditional foods, especially during childhood and adolescence. In non-diabetic subjects, the fasting insulin concentration decreased by 7% (95% CI –12 to –2%) for each doubling of the PCB concentration after adjustment for sex and BMI at age 20. Conversely, the fasting glucose concentration increased by 6% (–1 to 13%) for each doubling in PCB. Similar associations were seen in subjects without impaired fasting glycaemia, while further adjustment for current BMI and lipid metabolism parameters attenuated some of the associations. According to this study, impaired insulin secretion appears to constitute an important part of the type 2 diabetes pathogenesis associated with exposure to persistent lipophilic food contaminants (65).

## Type 2 diabetes in middle-aged Faroese residents

In total, 3,324 people were included in the Faroese Diabetes project. The individuals were derived from three different groups: 460 subjects with diabetes or prediabetes found in a cross-sectional population-based study (MARK) conducted in 2007–2008; 577 individuals from the septuagenarians cohort with increased or marginally elevated HbA1c or blood glucose levels; and a new group of 2,187 randomly selected individuals aged between 40 and 70 years.

About 26% of the study population had some carbohydrate disturbance. The overall prevalence of type 2 diabetes mellitus in the study population was 13.1% (95% CI 11.7–14.5%). Of these, about a quarter had unknown diabetes: 3.4% (2.7–4.1%). The prevalence of isolated impaired fasting glucose (IFG), isolated impaired glucose tolerance (IGT) and combined IFG–IGT was 4.8% (3.9–5.7%), 4.5% (3.7–5.4%) and 3.5% (2.8–4.3%), respectively. The prevalence of diabetes and other disorders increased significantly with age (p<0.05) and was higher in men than in women (p<0.0001). Age- and sex-specific prevalence of type 2 diabetes mellitus was 2.1% (6/284), 6.0% (16/266), 8.9% (20/225) and 18.8% (60/319) for women aged 40–49, 50–59, 60–69 and 70+, respectively, and 3.8% (12/313), 9.5% (27/283), 24.2% (64/264) and 28.3% (96/339), respectively, for men (68).

## Conclusions and recommendations

Maximizing the returns from such studies requires a harmonized study design and harmonized reporting of results. This will make it possible to merge studies and perform strong meta-analysis. AMAP guidance on the design of cohort and dietary studies for assessing the effects of environmental contaminants on population health in the Arctic as well as a protocol for the full reporting of results, including statistical methodology, would be very useful. This will enhance the ability to compare and combine the outcome of such studies from different circumpolar regions and should thus result in a more statistically valid assessment of effects. An increasing number of exposure studies have been performed in the circumpolar Arctic over the past three decades. Exposure in this context means exposure to contaminants in the Arctic environment. The main source of contaminant exposure is the consumption of traditional foods of marine origin, such as whales, seals, polar bears and some fish species. AMAP has generated a vast amount of data on contaminant levels in human tissues, especially in hair and blood, and in some studies even in human milk. Exposure levels vary in different regions of the Arctic, which can be largely explained by variation in contaminant levels in the traditional diet.

Several studies have been designed as birth cohorts, giving the opportunity for later examination of health effects associated with prenatal or early postnatal exposure. However, conducting human health effects studies in the Arctic can be challenging for several reasons, including issues associated with logistics, the wide range of languages and cultures, and a lack of qualified staff when estimating the function of the central nervous system.

Maximizing the returns from such studies requires a harmonized study design and harmonized reporting of results. This will make it possible to merge studies and perform strong meta-analysis. AMAP guidance on the design of cohort and dietary studies for assessing the effects of environmental contaminants on population health in the Arctic as well as a protocol for the full reporting of results, including statistical methodology, would be very useful. This will enhance the ability to compare and combine the outcome of such studies from different circumpolar regions and should thus result in a more statistically valid assessment of effects.
